# An Atomic Force Microscope Study Revealed Two Mechanisms in the Effect of Anticancer Drugs on Rate-Dependent Young’s Modulus of Human Prostate Cancer Cells

**DOI:** 10.1371/journal.pone.0126107

**Published:** 2015-05-01

**Authors:** Juan Ren, Huarong Huang, Yue Liu, Xi Zheng, Qingze Zou

**Affiliations:** 1 Department of Mechanical and Aerospace Engineering, Rutgers, the State University of New Jersey, Piscataway, NJ, 08854, USA; 2 Allan H. Conney Laboratory for Anticancer Research, Guangdong University of Technology, Guangzhou, 510006, P. R. China; 3 Department of Chemical Biology, Ernest Mario School of Pharmacy, Rutgers, the State University of New Jersey, Piscataway, NJ, 08854, USA; LAAS-CNRS, FRANCE

## Abstract

Mechanical properties of cells have been recognized as a biomarker for cellular cytoskeletal organization. As chemical treatments lead to cell cytoskeletal rearrangements, thereby, modifications of cellular mechanical properties, investigating cellular mechanical property variations provides insightful knowledge to effects of chemical treatments on cancer cells. In this study, the effects of eight different anticancer drugs on the mechanical properties of human prostate cancer cell (PC-3) are investigated using a recently developed control-based nanoindentation measurement (CNM) protocol on atomic force microscope (AFM). The CNM protocol overcomes the limits of other existing methods to in-liquid nanoindentation measurement of live cells on AFM, particularly for measuring mechanical properties of live cells. The Young’s modulus of PC-3 cells treated by the eight drugs was measured by varying force loading rates over three orders of magnitude, and compared to the values of the control. The results showed that the Young’s modulus of the PC-3 cells increased substantially by the eight drugs tested, and became much more pronounced as the force load rate increased. Moreover, two distinct trends were clearly expressed, where under the treatment of Disulfiram, paclitaxel, and MK-2206, the exponent coefficient of the frequency- modulus function remained almost unchanged, while with Celebrex, BAY, Totamine, TPA, and Vaproic acid, the exponential rate was significantly increased.

## Introduction

Mechanical properties of live cells are known to be closely related to the cells’ growth stage and functionality. Changes in mechanical properties have been recognized as an indicator of pathological modifications of cells [1–3] and thereby, can serve as a biomarker for cellular phenotypic events, for example, those associated with cell adhesion and cytoskeletal organization [4–6]. In particular, as a response to the environmental and/or mechanical condition variations, cell cytoskeleton undergoes dynamical rearrangements, which, in return, further induces changes to the cellular mechanical properties [7]. Therefore, studies of mechanical properties of cells contributes to a better understanding of cells’ responses to chemical treatments, including drug treatments of cancer cells. It has been reported that diseases such as cancers alter the mechanical properties of the cells [1, 8, 9], and reversely, variations of mechanical properties of cancer cell caused by anticancer drugs may be employed to evaluate the efficacy of these chemicals [10, 11]. Investigations of mechanical properties of cancer cells can further help to unravel the physical mechanisms involved in cancer development, progression and metastasis. Therefore, study of cellular mechanical properties becomes an indispensable and critical component in the development of novel strategies for cancer prevention and diagnosis.

Atomic force microscope (AFM) has become a powerful tool to study mechanical properties of single live cell owing to its unique capability in applying force stimuli and then, measuring the response at specific locations in a physiologically friendly environment with piconewton force and nanometer spatial resolutions [8, 12]. Specifically, AFM has been utilized to investigate the evolution of cell mechanical properties caused by cell abnormalities (e.g., cancers) and chemical treatments on cancerous cells [7, 8]. For example, it has been found that the Young’s modulus of cancerous human epithelial cells tend to be substantially lower than normal ones [3], the Young’s modulus of breast cancer cells increases monotonically with the increase of the force load rate [8], and after F-actin-disrupting drug treatment, the average elastic modulus of fibroblast cells decreased distinctly [10]. However, these studies [3, 8, 10] have been limited to measuring static cellular mechanical behavior in low frequency regions (with force load rate below 5 Hz) and small force amplitudes (below 2 nN). The dynamic mechanical behaviors of cancer cells in higher frequency regions, and the effects of chemical treatments on the frequency-dependent viscoelastic behavior of cancer cells are largely unknown. As chemical treatments lead to dynamical rearrangements of cell cytoskeleton, and thereby, dynamic evolution of mechanical properties of cells [7, 10], evolution of dynamic mechanical behaviors of cancer cells provide insightful information to anticancer drug development.

Studies of frequency-dependent biomechanical properties of live cells have been limited by the capability of current AFM mechanical measurement techniques. Specifically, the limit arises largely due to the current method to indentation measurement on AFM, by subtracting the cantilever deflection from the cantilever base displacement [8, 13]. Significant errors and uncertainties are induced in the indentation measured as the probe acceleration (with respect to the fixed-end of the cantilever attached to the piezoelectric scanner) is ignored and the initial contact point is largely uncertain [8, 13–15]. Particularly, the probe acceleration effect is pronounced and increases substantially when the measurement frequency increases. Although the force-modulation method has been employed to measure the frequency-dependent viscoelasticity of live cells [16, 17], by augmenting a sinusoidal oscillation to the load/unload force profile of constant rate, the probe acceleration effect is completely ignored, and large uncertainties exist in the indentation measured in the relatively high frequency region [14, 18]. Moreover, such an approach is further limited by the rather small amplitude of the dynamic force applied (tens of peco newtons) applied—whereas to interrogate a variety of biological responses of the cell, excitation force of much larger amplitude needs to be applied as the mechanical properties of live cells are amplitude dependent [19, 20]. Finally, the force modulation method requires the oscillatory force to be repetitively applied at the same location at each selected measurement rate in the measured frequency range. However, for live cells such a procedure is detrimental as the repetitive, same-location force exertion tends to deform and even damage the cell membrane. It was also proposed to study mechanical characteristics of live cell by quantifying the effective stiffness using a magnetic force modulation technique on AFM [15]. Such a method, however, not only requires additional sample/ equipment preparation (e.g., use of a home-built aluminum holder with vacuum grease to mount the sample), but also does not quantify the cell stiffness (i.e., the Young’s modulus) directly [15]—Quantification of the Young’s modulus requires accurate measurement of the indentation. Since the force stimuli applied and the corresponding indentation generated act as, respectively, the input and output to the cantilever probe-sample interaction model, error in indentation measurement leads directly to that in the mechanical property quantified—regardless the probe-sample interaction model employed. Thus, it is crucial to accurately measure the indentation in mechanical studies of live cell.

In this study, the effect of anticancer chemical compounds on the dynamic mechanical properties of human prostate cancer cell (PC-3) is investigated by using a newly developed control-based nanoindentation measurement (CNM) protocol [14]. Eight distinct drugs, including Disulfiram (DSF), paclitaxel (Taxol), Tomatine, BAY 11-7082 (BAY), vaproic acid (VPA), 12-O-tetradecanoylphorbol-13-acetate (TPA), Celecoxib, and MK-2206 (MK) are tested. Although studies have shown the anticancer effects of these drugs, for example, proteasome inhibition and apoptosis process of breast cancer cells induced by DSF—a well-known drug for alcoholism treatment [21], experimental examinations of these chemical compounds as anticancer drugs are ongoing, and many questions remain unanswered. Therefore, studying the dynamic mechanical property changes of PC-3 cells treated by these eight drugs may provide insightful answers to the anticancer activities of these chemical compounds.

The CNM protocol [14] overcomes the limits of existing methods for in-liquid indentation measurement of soft samples on AFM. By the CNM protocol, the indentation on the live cell is measured by tracking the *same* excitation force profile (i.e., the same cantilever deflection) on both the live cell and a hard reference, and then quantified from the displacement difference of the cantilever fixed end on these two samples. The main advantage of the CNM protocol is that by using a hard reference and more critically, accurately tracking the same force profile on both the samples, the dominant adverse effect of the cantilever acceleration is completely removed with no need for parameter calibration [14, 18]. Moreover, the hydrodynamic force effect is substantially reduced, particularly at high force load rates (e.g., reduced by over 50% when the force load rate is higher than 100 Hz). In this study, the rate dependent Young’s modulus of PC-3 cells was quantified using the CNM protocol by varying the load/unload rate of the excitation force over three orders of magnitude from 0.2 Hz to 100 Hz, with the measured indentation amplitude over 2 orders larger than the oscillatory amplitude in [16].

## Materials and Methods

### Cell culture and treatment

PC-3 cells were obtained from the American Type Culture Collection (ATCC, Rockville, MD, USA), and grown in RPMI-1640 culture medium containing 10% FBS that was supplemented with penicillin (100 units/ml)-streptomycin (100 *μ*g/ml) and L-glutamine (300 *μ*g/ml). Cells were cultured at 37°C in a 5% CO_2_ incubator and passaged twice a week. To accommodate the AFM measurements, the PC-3 cells were seeded at a density of 2.0 × 10^4^ cells/ml in 60 mm tissue culture dishes (5 ml/dish) and incubated for 24 hpurs. Then the cells in each dish were then treated with solvent DMSO (2 *μ*l/ml) or with each of the eight drugs dissolved in DMSO for 24 hours before the AFM measurements.

### MTT assay

PC-3 cells were seeded at a density of 2.0 ×10^4^ cells/ml of medium in 96-well plates (0.2 ml/well) and incubated for 24 h. The cells were then treated with the various anticancer agents for 72 h. After treatment, 200 *μ*l 3-[4,5-dimethylthiazol-2-yl]-2,5-diphenyl tetrazoliumbromide (5 mg/ml in PBS) was added to each well of the plate and incubated for 2 h. After careful removal of the medium, 0.1 ml DMSO was added to each well. The absorbance was recorded on a microplate reader at 540 nm. The effect of different anticancer agents on cell viability was assessed as viability percentage as compared to the DMSO-treated cells.

### Immunofluorescence

Immunofluorescence staining was used to determine *β*-actin in PC-3 cells. Briefly, PC-3 cells were seeded at a density of 2.0 ×10^4^ cells/ml of medium in 60 mm culture dishes and incubated for 24 h. The cells were then treated with MK or Celebrex for 24 h. Afterwards the cells were fixed in acetone/methanol (1:1) for 10 min at room temperature and then incubated with *β*-actin antibody (sc-47778, Santa Cruz Biotech Inc, Dallas, TX) overnight at 4°C. Next the cells were washed and incubated with Texas Red conjugated goat anti mouse antibody (115-075-003, Jackson ImmunoRsearch Lab Inc, West Grove, PA) for 60 min at room temperature. Immunofluorescence staining was examined using a fluorescence microscope (Nikon Eclipse TE200, Nikon Inc.).

### Chemicals

The RPMI-1640 tissue culture medium, penicillin-streptomycin, L-glutamine and fetal bovine serum (FBS) were acquired from Gibco (Grand Island, NY). Among the eight different drugs tested in this study, Disulfiram (DSF), paclitaxel (Taxol), tomatine, BAY 11-7082 (BAY), vaproic acid (VPA), and 12-O-tetradecanoylphorbol-13-acetate (TPA) were acquired from Sigma-Aldrich (St. Louis, MO), and Celecoxib and MK-2206 (MK) were provided by the National Cancer Institute’s Repository.

### Control-based Elasticity Measurements

The recently-developed CNM protocol [14, 18, 22] was employed to measure the rate-dependent Young’s modulus and frequency-dependent complex modulus of EA.hy926 cells. The central issue is to measure the indentation in the live cell accurately, particularly during high-speed and/or broadband nanomechanical measurements. Based on the analysis of the cantilever dynamics during the nanoindentation measurement, the CNM protocol obtains the indentation in the live cell, Δ_*z*_(*t*), as the difference of the displacement of cantilever base (i.e., the fixed end of the cantilever) on the cell, *z*_*bs*_(*t*), and that on a hard reference sample (e.g., a silicon sample), *z*_*bh*_(*t*) [14],

$$\begin{array}{c} \Delta_{z}\left(t\right)=z_{bs}\left(t\right)-z_{bh}\left(t\right). \end{array}$$ (1)

The above indentation quantification requires that the *same* excitation force profile (i.e., the same cantilever deflection trajectory) is tracked accurately on both the samples. The readers are referred to Ref. [16] for details of the CNM protocol.

To ensure precision tracking of the same excitation force profile on both the live cell and the hard reference, the CNM protocol utilizes iterative learning control techniques, for example, the modeling-free inversion-based iterative learning control (MIIC) technique [23]. Specifically, the control input applied to drive the AFM *z*-axis piezo actuator is obtained through iteration as follows:

$$\begin{array}{ccc} u_{1}\left(j\omega \right) & = & \alpha d_{d}\left(j\omega \right),\;k=1,\\ u_{k+1}\left(j\omega \right) & = & \left\{ \begin{array}{cc} \frac{u_{k}\left(j\omega \right)}{d_{k}\left(j\omega \right)}d_{d}\left(j\omega \right), & \text{when}\;d_{k}\left(j\omega \right)\neq 0\;\text{and}\;d_{d}\left(j\omega \right)\neq 0,\;k\geq 1,\\ 0, & \text{otherwise} \end{array}\right. \end{array}$$ (2)

where‘*jω*’ denotes Fourier transform. *d*_*d*_(⋅) is the desired cantilever deflection, *α* is a constant, and *u*_*k*_(⋅) and *d*_*k*_(⋅) are the current input voltage to the AFM piezo actuator and the cantilever deflection in the *k* ^*th*^ iteration, respectively. The control input *u*_*k*_(*t*) is obtained by taking Fourier transform of the input and the output signals and applying the MIIC algorithm Eq 2, and then the inverse Fourier transform afterwards. The MIIC algorithm has also been utilized to obtain rapid broadband nanomechanical measurement on polymers in air recently [24, 25].

### Atomic force microscope

Young’s modulus of the PC-3 cells was measured in the cell culture medium using a Dimension Icon AFM (Bruker, Santa Barbara, CA) equipped with a fluid cell. A soft cantilever (MLCT-C, Bruker, USA) with nominal spring constant 0.01 N/m was chosen for the measurements. The probe radius of 28 nm and the cantilever spring constant of 0.012 N/m were calibrated, respectively, by imaging a tip-radius calibration sample (PA-01, Mikromasch, NanoAndMore USA Corp.) and the thermal tuning process. A silicon sample was chosen as the hard reference sample. Both the cells and the cantilevers were thermally equilibrated at ∼ 37°C for 40-60 mins prior to all measurements to minimize the cantilever drifts. All of the control and sensor signals to/and from the AFM system were acquired through a data acquisition system (NI PCI-6259, National Instruments Corporation, Austin, TX) under the Matlab xPC-target (The MathWorks, Natick, MA) environment.

A triangle drive voltage with a constant load and unload rate (as employed in usual force-distance curve measurement) was applied to the *z*-axis piezo actuator of the AFM system, and the following nine load rates over three orders of magnitude were applied: 0.2 Hz, 0.5 Hz, 1 Hz, 5 Hz, 10 Hz, 20 Hz, 50 Hz, and 100 Hz. The amplitude of the drive voltage was kept the same for all the above load rates, resulting in the same cantilever base displacement at 250 nm (as for the above load rates, the dynamics of neither the *z*-axis piezoelectric actuator nor the cantilever fixture mechanism was excited [18]). To minimize the cell membrane damage, the triangle drive was applied for only one period when the force load rate was lower than 50 Hz and two periods at higher load rates. The drive inputs were applied successively from low to high load rates, separated by a dwelling time of 3 min between each rate—to allow the cell to fully recover from the preceding force stimuli. For each load rate, the excitation force exerted (i.e., the cantilever deflection) on the live cell was measured and regarded as the desired excitation force profile, and the MIIC algorithm was applied in the force-distance curve measurement on the reference sample to ensure precision tracking of the desired force profile (the RMS tracking error was maintained below 1.5%).

To study the effect of each drug on Young’s modulus of PC-3 cells, the measurements were performed on the corresponding control first, then on the treated cells. For each drug, these measurements were repeated on five different cells for both the control and the drug treated ones.

### Rate-dependent Elastic Modulus Quantification

At each force load rate, the Young’s modulus of cell was quantified using the spherical Hertzian contact model along with the measured probe-sample interaction force and indentation [12],

$$\begin{array}{c} F_{z}=\frac{4}{3}\frac{E\sqrt[{}]{R\Delta_{z}^{3}}}{1-\nu^{2}}, \end{array}$$ (3)

where *R* is the probe radius, and *E* and *ν* are the Young’s modulus and the Poisson ratio of the live cell (*ν* = 0.5 [7, 12]), respectively. The probe-sample interaction force is quantified as *F*_*z*_ = *k*_*c*_ *d*_*s*_ (with cantilever spring constant (*k*_*c*_)) [12]. We note that other Hertizan indentation contact model such as the conical model [12] might be used. The spherical contact model is chosen as in this work the indentation depths generated were not substantially larger (over 10 times) than but tend to be comparable to the probe radius [26, 27].

## Results and Discussion

**Fig 1:**
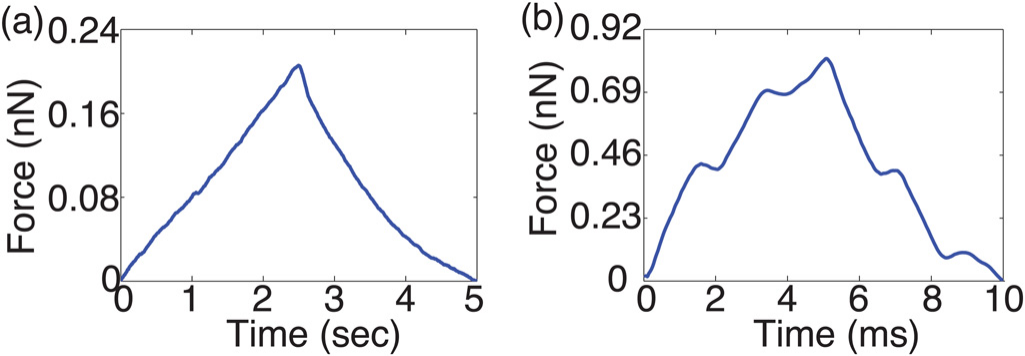
Force time profile on 20 *μ*M TPA treated cells at the load rate of (a) 0.2 Hz, and (b) 100 Hz.

**Fig 2:**
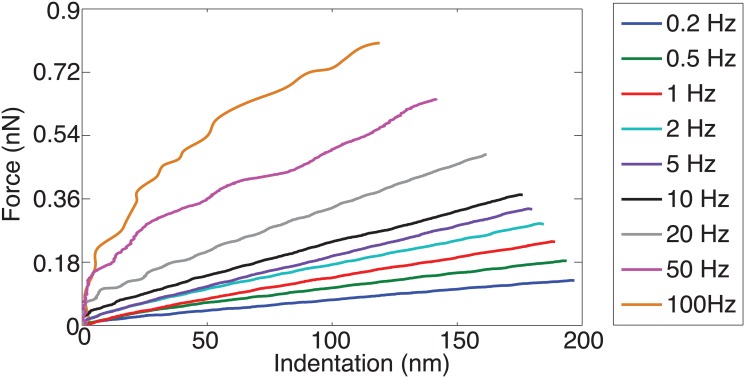
Force vs. indentation curve measured on 20 *μ*M TPA treated cells.

**Fig 3:**
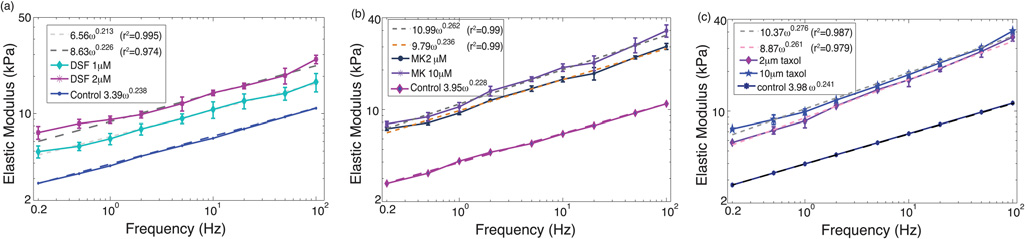
Young’s modulus of PC-3 cells treated by: (a) DSF, (b) MK, and (c) Taxol, respectively.

**Fig 4:**
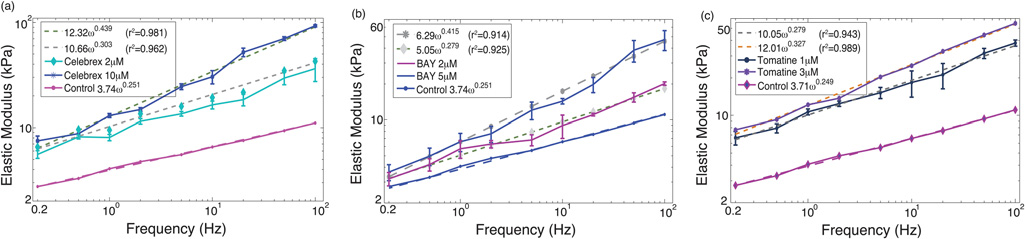
Young’s modulus of PC-3 cells treated by: (a) Celebrex, (b) BAY, and (c) Totamine, respectively.

**Fig 5:**
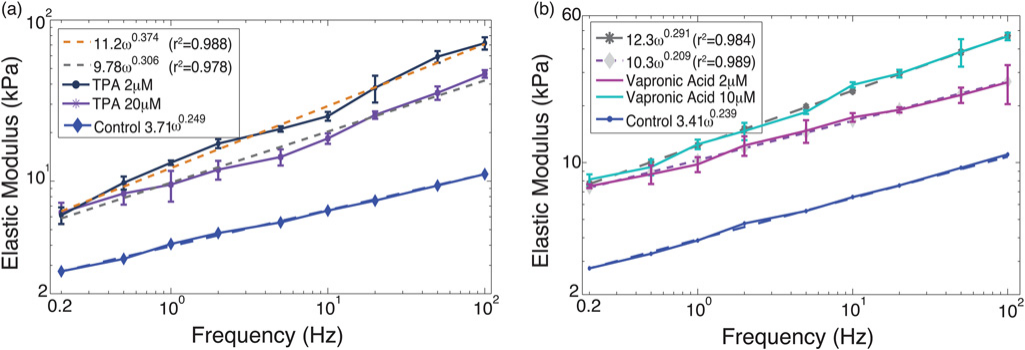
Young’s modulus of PC-3 cells treated by: (a) TPA, and (b) Vapronic-Acid, respectively.

**Fig 6:**
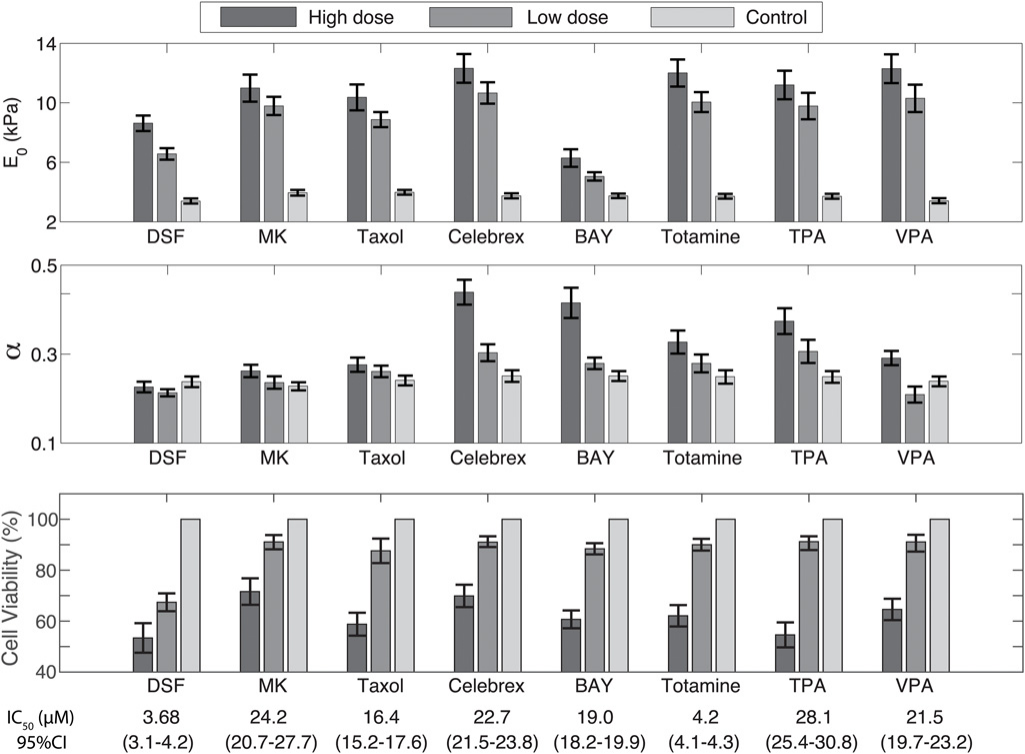
Comparison of *E*_0_ (kPa) and *α* in the power-law relation (Eq 4), and cell viability determined by the MTT assay for the eight drugs of both low and high dosages, compared to the corresponding controls.

The force (i.e., cantilever deflection) time profile at the load rates of 0.2 Hz and 50 Hz on TPA treated PC-3 cells at high dosage (20 *μ*M) is shown in Fig 1, as an example—the force-time plots of the low dosage and/or other drugs showed similar trend. The force-indentation curves measured from the same treatment at all nine load rates are shown in Fig 2 for all the nine load rates (force-indentation curves for other measurements are not shown to save sapce). The Young’s modulus of the control (i.e., untreated) and the drug treated PC-3 cells are compared in Figs 3–5 for the eight tested drugs, respectively, where the Young’s modulus vs. the force load rate is plotted in logarithmic scale, and the curve-fitting of the data to the following power law is also shown,

$$\begin{array}{c} E=E_{0}\omega^{\alpha }, \end{array}$$ (4)

where *E*_0_ is the power law constant–the elasticity scale factor of cells, and *α* is the power law exponent that captures the viscosity of the cell membrane [13, 28]. Moreover, *E*_0_ and *α* of the fitted Young’s modulus vs. frequency curve are also compared in Fig 6 for the eight drugs tested for the control and the treated PC-3 cells under both the low and the high dosages.

The experiment results showed that the viscoelastic behaviors of the PC-3 cells were well captured in this work. As shown in Fig 1(b), the probe acceleration effect on the force-indentation trajectory was pronounced and needed to be accounted for in the indentation quantification, and for the same driven amplitude, the indentation generated decreased monotonically with the increase of the force load rates (see Fig 2), reflecting the viscoelasticity nature of the cell membrane [8, 12]. The indentation generated on the PC-3 cells ranged from 80 nm to 230 nm among all the eight tested drugs (for all the tested dosages). As the *z*-axis driven driven amplitude was kept the same at 250 nm, such a variation of the indentation exactly reflected the viscoelasticity of the cells and the drug effects on it, respectively.

The measured Young’s modulus vs. frequency relation followed, quite well, the power law—the widely observed universal viscoelastic behavior of live human cells [13, 28, 29]. The variations of the elasticity scale factor *E*_0_ and the power law exponent *α* were small among all the controls—with the standard deviation at 5.8% and 4.2%, respectively (see Fig 6), respectively. Such a small variation of *E*_0_ and *α*, therefore, can be served as the baseline to examine the effects of the nine tested drugs on the mechanical properties of the PC-3 cells. Moreover, the range of the power law exponent *α* agrees well with the reported range (0.1–0.3) for live cells in literature [29].

As shown in Figs 3–5, all of the drug treated cells presented a much higher Young’s modulus, and the higher the drug dosage was, the larger the increase of Young’s modulus was. As the Young’s modulus change in cells is directly related to remodeling of the cytoskeletal structure [4, 5, 10], one possible explanation of the modulus increase may be the aggregation of actin filaments under the drug effects since it has been shown that aggregation of actin filaments results in a distinct increase in the average Young’s modulus of cells [10].

A quick comparison of Figs 3–5 revealed two major trends might exist in the eight test drugs effects on the Young’s modulus: under the effect of DSF, MK, and Taxol, the Young’s modulus of PC-3 cells was increased without significant changes in the frequency-dependence, i.e., the elasticity scale factor *E*_0_ increased substantially (by 55% to 78%), while the power law exponent *α* remained almost unchanged (see Eq 4)—for these drugs the variation of *α* was only about 6% to 14%. Whereas under the effect of Celebrex, BAY, Totamine, TPA, and VPA, the frequency-dependency of the elevated Young’s modulus changed significantly, i.e., *E*_0_ increased by 78% to 260%, while *α* also increased by 22% to 75% (see Fig 6).

### DSF, MK and Taxol: Elevated Young’s Modulus without Significant Change of Frequency-dependency

For DSF, MK, and Taxol, the ratios of the Young’s modulus between the treated PC-3 cells and the control were nearly the same cross all nine measured frequencies at each treatment dosage (shown as the increase of *E*_0_, see Fig 3). This implies that the viscosity of the treated cells was not changed substantially compared to the control ones as the value of *α* didn’t change substantially. It can be concluded that under the treatment of DSF, MK and Taxol, the cell cytoskeleton network reconstruction may lead to an overall stiffening of the membrane protein structure (e.g., filament shortening and thickening), but may not cause much change of degree of polymerization of actin filaments inside the cells—the general cause of viscosity change [30, 31].

Although MK, Taxol and DSF may have distinct molecular targets and mechanisms of action, the similar trend of cell Young’s modulus change may explain the similarity of these three drugs’ effects in cell mechanical behavior. MK can inactivate Ezrin which serves as linkers between plasma membrane and cytoskeleton [32]. Taxol interferes with normal breakdown of microtubules [33], and DSF inhibits tubulin polymerization [34]. It is reasonable to postulate that interfering with linkers between plasma membrane and cytoskeleton, interfering with microtubules breakdown and inhibiting tubulin polymerization could lead to cell stiffening without alteration of viscosity.

However, the Young’s modulus increase on MK and Taxol treated cells was more significant (even for a low dosage of 2 *μ*M) than that for DSF treated cells (at the same dose). One possible explanation may be the iron chelating effect of DSF. Earlier study showed that DSF facilitated intracellular Cu uptake [35]. It was found that MK and Taxol strongly inhibited activation of Akt [36, 37] while DSF had no inhibitory effect on activation of this protein [38]. Inactivation of Akt may contribute to the stronger effect of MK and Taxol as compared to DSF. The influence of DSF on iron homeostasis may be another possible explanation for the weaker effect of DSF (on the increase in Youngs modulus) than MK and Taxol.

### Elevated Young’s Modulus Accompanied by Dramatic Frequency-dependency Change

Strikingly different from the above three drugs, the other five drugs (Celebrex, BAY, Totamine, TPA, and VPA) tended to effect not only the elastic but also the viscous behavior of the PC-3 cells as both the elasticity scale factor, *E*_0_ and the power law exponent, *α*, were increased significantly. This phenomenon indicates that the cell cytoskeleton change due to the treatment of these five drugs consists not only cytoskeleton stiffening but also change of degree of polymerization, which may involve an increased concentration of actin monomers and a reorganization of actin filaments. Moreover, it is noted that the standard deviation of the Young’s modulus of the PC-3 cells treated by these five drugs are larger than that of those treated by the other three drugs (DSF, Taxol and MK). Since the standard deviation of the Young’s modulus for all control remains much smaller for all the eight drugs, one possible explanation for the larger deviation is that the dynamic mechanical behavior of the cells treated by the five drugs (Celebrex, BAY, Totamine, TPA, and VPA) was more active.

It was known that celebrex, TPA and valproic acid induced cell differentiation [39–41]. As studies showed that cell differentiation leads to an increase of cell rigidity [26], increase in stiffness and viscosity of PC-3 cells treated with celebrex, TPA and valproic acid may be related to the differentiation of the cells. Although BAY11-7082 and Tomatine have not been shown to induce differentiation in epithelial cells, these drugs inhibit activation of NF-*κ*B [42], and inhibition of NF-*κ*B in some cells resulted in a more differentiated phenotype [43]. Thus, the effects of BAY11-7082 and Tomatine on stiffness and viscosity of PC-3 cells may relate to their inhibitory effect on activation of NF-*κ*B.

Among the measurement results, the change of Young’s modulus was most significant on the Celebrex treated cells. Since Celebrex causes loss of filopodia and lamellipodia in cells, and changes in actin network [44], these activities in addition to its differentiation inducing effects may result in a stronger effect on increasing stiffness and viscosity of PC-3 as compared to the other four drugs.

We further performed MTT test to investigate the correlation between the changes of mechanical properties and the inhibition of cell growth. As shown by the cell viability test results from the MTT assay in Fig 6, treatment of PC-3 cells with the eight anticancer drugs decreased the number of viable cells, particularly at high dosage, i.e., the effect was dose-dependent. Such an effect of the drugs (on decreasing cell viability) correlated well with their effect on changing the cell mechanical properties revealed in the above AFM tests, for all eight different drugs examined. Although the above AFM studies clearly revealed the two distinct patterns among the eight different drugs in changing the mechanical properties of PC-3 cells (DSF, MK and Taxol as one group, and Celebrex, Bay, Tomatine, TPA and Vaproic acid as the other group), the MTT assay failed to show any evident difference in cell viability changes between these two groups. This result suggests that the proposed AFM studies might have revealed new aspects of biological response of cancer cells to anticancer drug treatments, thereby, providing more information than the conventional MTT assay in responses of PC-3 cells to anticancer drug treatment. One possible explanation is that the changes in cytoskeleton and cell membrane may correlate with the ability of cancer cells to metastasize. Future studies with appropriate cancer cell metastasis model may help to explore the correlation between changes in mechanical properties and metastatic ability of cancer cells.

**Fig 7:**
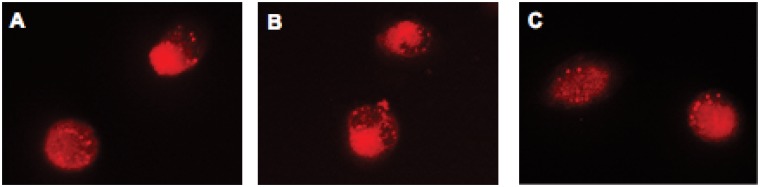
Comparison of the immunofluorescence images of (a) control, (b) cells treated with MK, and (c) cells treated with Celebrex.

To further investigate the mechanisms behind the two patterns revealed by the AFM studies, immunofluorescence staining of *β*-actin was conducted to seek insights to the different responses in PC-3 cells to the drug treatments. MK and Celebrex were selected to represent the first and the second group of the eight drugs (as revealed by the AFM studies), respectively. The fluorescence imaging results obtained are shown in Fig 7. The morphology of *β*-actin immunofluorescence staining was similar in cells treated with MK and those treated with Celebrex. This result suggests that the different responses in PC-3 cells to the two groups of drugs may involve more complex mechanisms in addition to the modification of the cell actin. Further studies on modification of other cytoskeleton components are needed to determine the mechanisms behind the two distinct types of response to the two groups of drugs tested.

The significance of the above studies is underscored by the importance of identifying new targets for inhibiting the growth and inducing apoptosis in cancer cells, which has become a major focus in the development of new generation of anticancer drugs. Physical properties of cancer cells such as elasticity and viscosity that associated with modification of cytoskeleton and plasma membrane may represent a unique class of novel target for development of anticancer drugs. Studies on alterations of physical properties in cancer cells treated with anticancer agents that have different mechanisms of action may provide insights to the identification of molecular targets causing lethal changes in physical properties of cells.

## Conclusion

In this study, the drug effect on cancer cell nanomechanical property change was investigated using the recently proposed CNM protocol. The Young’s modulus of PC-3 cells treated by eight different drugs was measured with force loading rates spanning three orders of magnitude, and compared to the values of the control. The results showed that the Young’s modulus of PC-3 cells were significantly increased by the eight drugs test, and became substantially more pronounced as the force load rate increased. Moreover, two distinct trends were clearly presented, where with DSF, Taxol, and MK, the exponent coefficient of the frequency-modulus relation remained almost unchanged, while under the effect the other five drugs, the exponential rate itself was substantially increased. These two trends pointed to the existence of two distinct mechanisms among these drugs in affecting the mechanical behavior of cancer cells, where the first group of drugs caused the cell cytoskeleton network reconstruction and might have led to the stiffening of the overall membrane protein structure (e.g., filament shortening and thickening), while the second group of drugs, in addition to causing the cytoskeleton network reconstruction, might have also changed the degree of polymerization of actin filaments inside the PC-3 cells. As a frequency-resolved Young’s modulus measurement provides deep insights into the cellular dynamics in response to changes of the chemical and mechanical environment, the results presented in this study indicate that nanomechanical property changes may be used as a novel determinant for screening and developing new anticancer agents.
